# 

**Published:** 1892-12

**Authors:** 


### LITERARY.

Thanks for the following :—

DISEASES OF THE LUNGS, HEART AND KIDNEYS. By Prof. N. S. Davis, Jr., A. M., M. D. The F. A. Davis Co., Philadelphia, publishers, 1231 Filbert street. 12 mo; 359 pp.; extra cloth, $1.25 net.

The above entitled book, like the generality of medical works published by the F. A. Davis Co., is a most exhaustive treatise of the several organs embraced in its title. One excellent feature of the book is its disregard of technical terms, which renders the text plain and comprehensive to the common reader, whilst to the medical practitioner, it offers a manual of great value, covering as it does, early-stage symptoms and prescribing the best mode of treatment to effect a cure known to science. No physician in active practice can afford to be without this handy little volume. The index is simply perfect, a feature of great concern for hasty reference.

ALL AROUND THE YEAR.—This is the title of an exquisitely illustrated calendar for the incoming new year from the publication house of Lee & Shepard, Boston, which has become noted for issuing yearly calendars of unrivalled beauty. The one in question is not a whit behind its predecessors in design and execution, and this is praise enough. The several monthly designs, from January to December, are on cards measuring 4½x5½ inches, and the whole are bound in silver rings and laced by a silken cord. Price, 50 cents.

JENNESS MILLER ILLUSTRATED MONTHLY is a most valuable addition to the journalistic literature devoted chiefly to the ways and wants of women. The

November number contains a well executed likeness of Mrs. Francis Hodgson Burnett, so well and favorably known as the author of "Little Lord Fountleroy."

AN OPERATION FOR CURE OF STRICTURE, Etc.; with description of a Stricturatome. By Charles Harmon Thomas, M. D., Philadelphia.

THE INFLUENCE OF FEAR IN DISEASE; by Dr. Wm. H. Holcombe, Chicago. Second edition ; price, 10 cents.

This is a valuable pamphlet of 13 pp. and containing much useful information and many useful suggestions, especially for the benefit of persons in charge of the sick and afflicted.

---

EDITOR HALL'S JOURNAL OF HEALTH.

Can you give me the ingredients of camphor ice and the mode of compounding them?—L. C. H.

Camphor ice is exceedingly hard, and the addition of an increased quantity of spirit of camphor is not calculated to soften it. I will give a few recipes for ointments which are made the vehicles of various drugs—such as resin, savin and camphor. Any amount of plain camphor up to one-twelfth part may be added. In camphor ice, however, the camphor does not seem to play an important part, because only a few drops of spirit of camphor are used; and of that drug only one part in ten is camphor, the rest being rectified spirit. The following is the "simple ointment" of the Pharmacopœia : Take 2 oz. of white wax, 3 oz. of benzoated lard, and 3 fluid ounces of almond oil; melt the wax and lard in the oil on a water-bath (which is never hotter than 212 degrees F.); remove the mixture and stir till cool. Another plan is to melt together 5 oz. of spermaceti, 2 oz. of white wax, a pint of almond oil. If half an ounce of benzoin be added to this, it becomes ordinary spermaceti ointment. But if half an ounce of plain camphor be added instead of the benzoin, it will be just what is now desired. A third recipe shall suffice for the present. Take 1 lb. of almond oil and 4 oz. of white wax; melt and gradually add a pint of rose water. This is "cold cream," otherwise called *unguentum galeni*, and a few drops of spirit of camphor would be a suitable addition. It is clear from the foregoing prescriptions that white wax and spirit without oil would be much too hard to be manageable.

